# Supplementary material for: Uncovering the roles of dihydropyrimidine dehydrogenase in fatty-acid induced steatosis using human cellular models
Source: Sci Rep. 2022 Aug 18;12:14109. doi: 10.1038/s41598-022-17860-2 (PMC9388600; doi:10.1038/s41598-022-17860-2)
Supplement: Supplementary file 1 — Supplementary Information. [file 41598_2022_17860_MOESM1_ESM.docx]

**Supplementary Information File**

**Title**: Uncovering the Roles of Dihydropyrimidine Dehydrogenase in Fatty-Acid Induced Steatosis using Human Cellular Models

**Authors**: Kelly E. Sullivan, Sheetal Kumar, Xin Liu, Ye Zhang, Emily de Koning, Yanfei Li, Jing Yuan, and Fan Fan

**Supplemental Table 1.** The nucleic acid sequences of the two gene block fragments used in molecular cloning of DPYD.


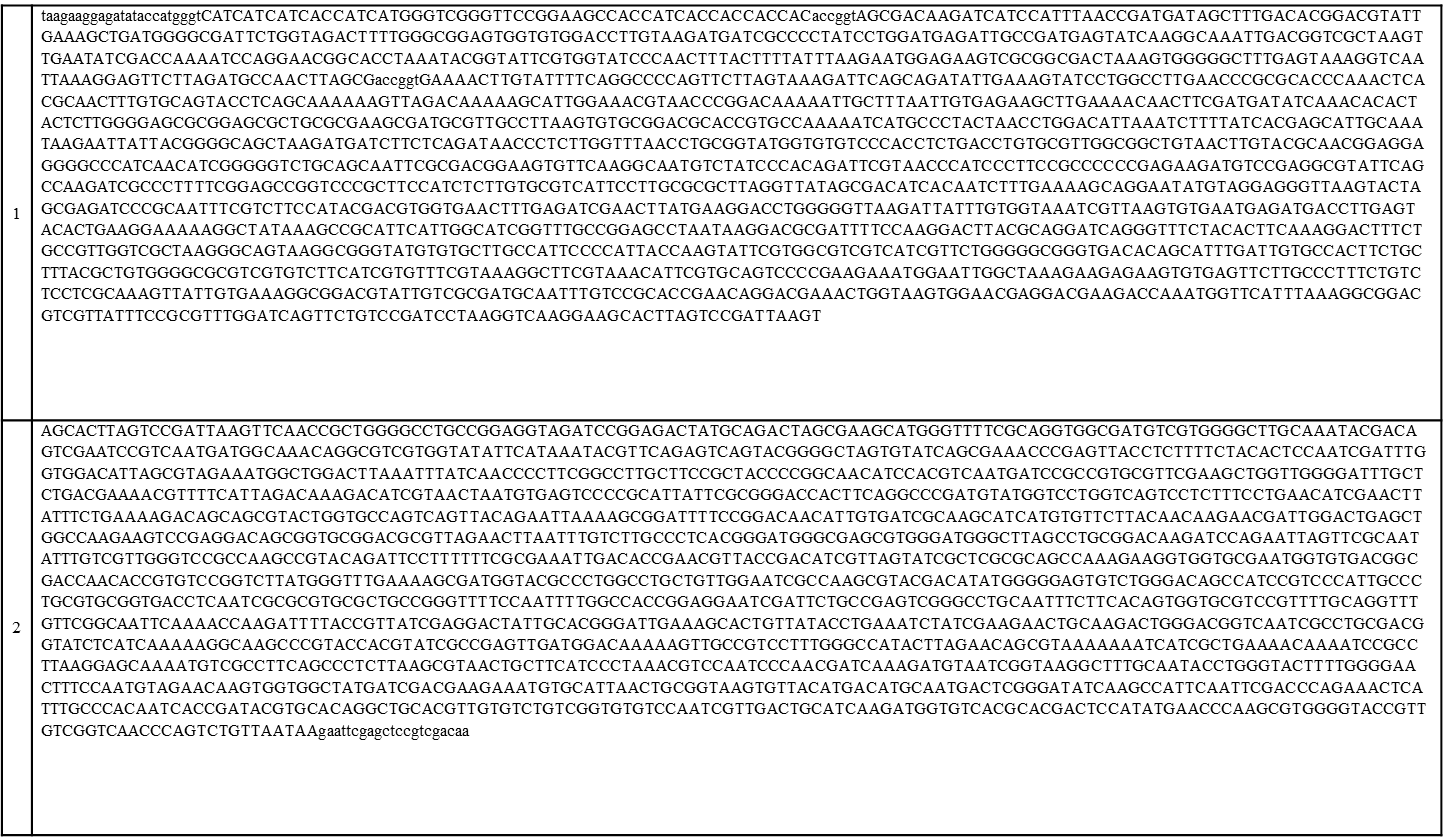


**Supplemental Table 2.** Sequences of first and second round primers used for next generation sequencing libraries.


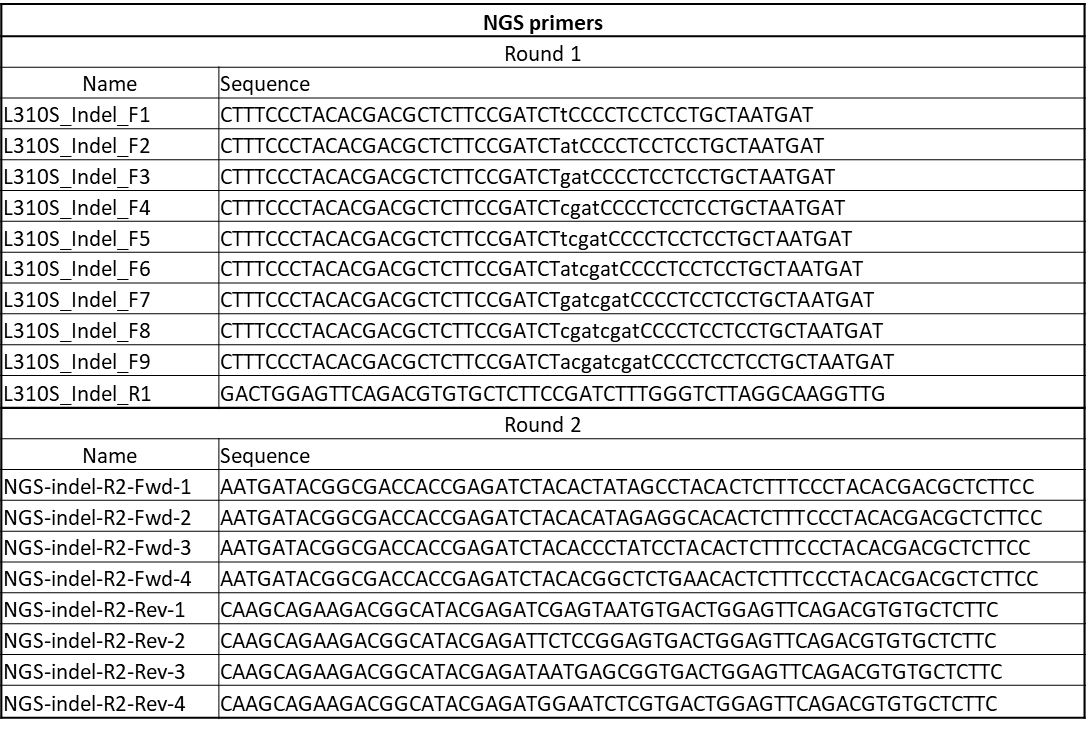


**Supplemental Table 3.** PCR primers used in qPCR and ddPCR, respectively.


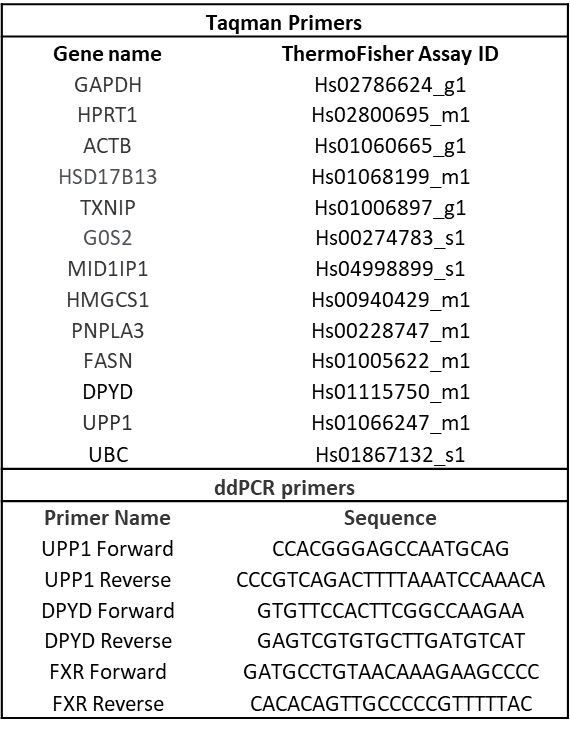


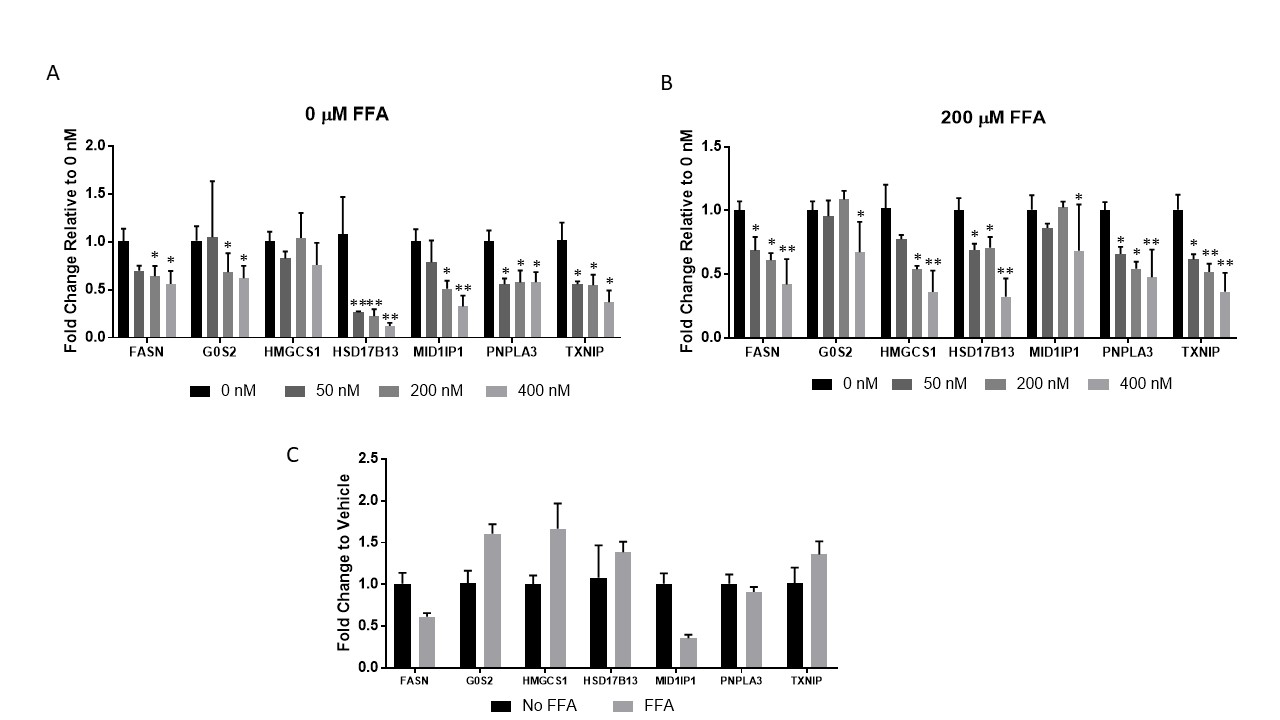


**Supplemental Figure 1.** Gene expression changes in SNU449 control line exposed to free fatty acids relative to no treatment. There is no significant change in expression of DPYD, FASN, G0S2, HMGCS1, HSD17B13, MID1IP1, PNPLA3 or UPP1. There is a significant increase in expression of TXNIP following free fatty acid treatment relative to no treatment (adjusted p-value <0.0001).

**Supplemental Figure 2.** Gene expression changes in SNU449 control line exposed to free fatty acids relative to no treatment. There is no significant change in expression of DPYD, FASN, G0S2, HMGCS1, HSD17B13, MID1IP1, PNPLA3 or UPP1. There is a significant increase in expression of TXNIP following free fatty acid treatment relative to no treatment (adjusted p-value <0.0001).


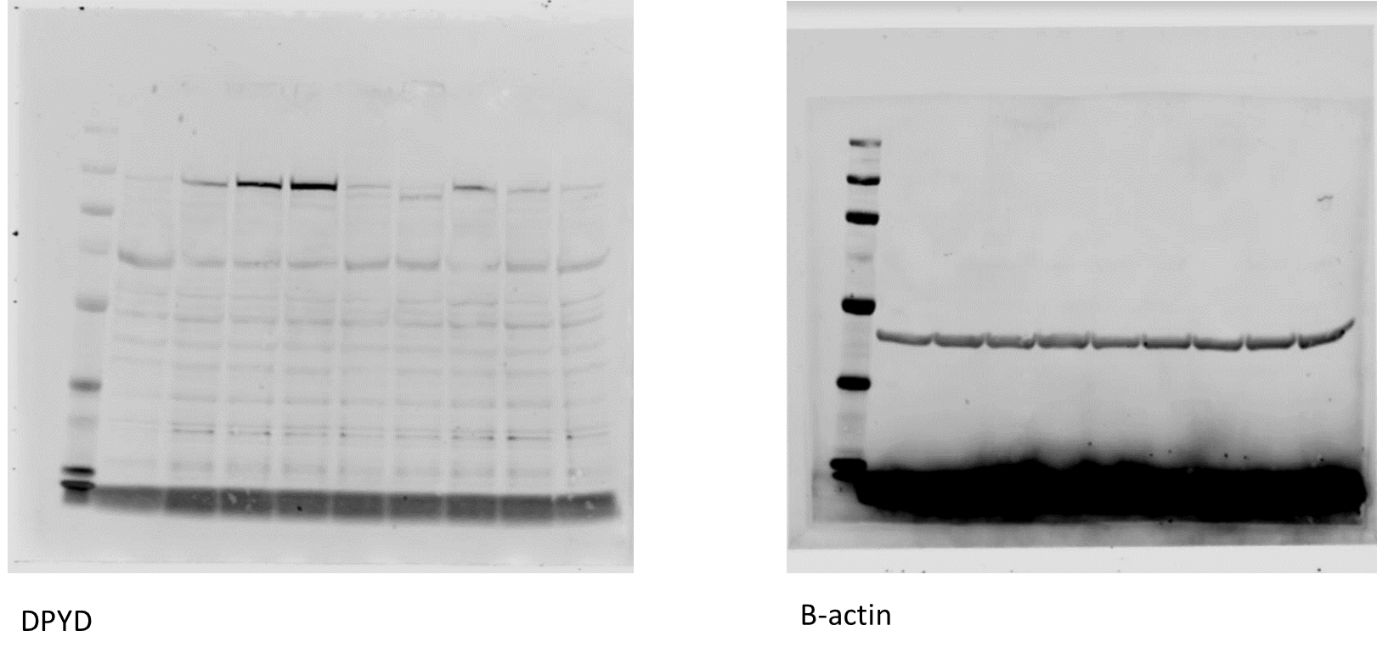


**Supplemental Figure 3.** Full gel images for Figure 7C. Both images were acquired from the same blot using a Licor imager and Dylight 680 and 800 secondary antibodies. Note the first lane is ladder and the next three lanes include cell lysate from clonal lines described in the manuscript. Additional lanes (5-10) describe lysate extracted from additional clonal lines not described in the manuscript.
